# Plasmonic Sensors Based on a Metal–Insulator–Metal Waveguide—What Do We Know So Far?

**DOI:** 10.3390/s24227158

**Published:** 2024-11-07

**Authors:** Muhammad A. Butt

**Affiliations:** Institute of Microelectronics and Optoelectronics, Warsaw University of Technology, Koszykowa 75, 00-662 Warsaw, Poland; ali.butt@pw.edu.pl

**Keywords:** plasmonics, metal–insulator–metal waveguide, surface plasmon polariton

## Abstract

Metal–insulator–metal (MIM) waveguide-based plasmonic sensors are significantly important in the domain of advanced sensing technologies due to their exceptional ability to guide and confine light at subwavelength scales. These sensors exploit the unique properties of surface plasmon polaritons (SPPs) that propagate along the metal–insulator interface, facilitating strong field confinement and enhanced light–matter interactions. In this review, several critical aspects of MIM waveguide-based plasmonic sensors are thoroughly examined, including sensor designs, material choices, fabrication methods, and diverse applications. Notably, there exists a substantial gap between the numerical data and the experimental verification of these devices, largely due to the insufficient attention given to the hybrid integration of plasmonic components. This disconnect underscores the need for more focused research on seamless integration techniques. Additionally, innovative light-coupling mechanisms are suggested that could pave the way for the practical realization of these highly promising plasmonic sensors.

## 1. Introduction

Plasmonics, the study of the interaction between an electromagnetic (EM) field and free electrons in a metal, has its roots in the early 20th century when the phenomenon of surface plasmon resonance (SPR) was first observed [1]. The foundational theoretical framework was laid by Mie in 1908 [2], who described the scattering of EM waves by spherical particles, and this was later refined by Ritchie in 1957 with the concept of surface plasmons on thin films [3]. The field gained significant momentum in the 1980s and 1990s with advancements in nanofabrication techniques, enabling the manipulation of light at the nanoscale. This period saw the development of various plasmonic devices such as sensors, waveguides (WGs), and more recently, plasmonic circuits, which promise to revolutionize fields like photonics and telecommunications [4]. Key milestones include the demonstration of extraordinary optical transmission through subwavelength hole arrays by Ebbesen et al. in 1998 [5] and the development of the first plasmonic circuits in the early 2000s [6,7,8]. Today, plasmonics is a vibrant field of research with applications ranging from high-resolution imaging and cancer therapy to advanced optical computing and beyond [9,10,11].

Plasmonic WGs exploit surface plasmon polaritons (SPPs) to confine and guide light at subwavelength scales [12]. These structures are pivotal in the field of nanophotonics because they allow for the manipulation of light below the diffraction limit, which is crucial for the progress of miniature and efficient photonic devices. SPPs are EM waves that travel along the interface between a metal and a dielectric, tightly bound to the surface due to the collective oscillation of electrons at the metal surface [13]. This unique property enables plasmonic WGs to achieve high field confinement, which is essential for various practicalities such as sensing, signal processing, and energy harvesting. Plasmonic WGs come in several configurations, including metal–insulator–metal (MIM) WGs [14], insulator–metal–insulator (IMI) WGs [15], and dielectric-loaded plasmonic WGs. Each configuration has its advantages and is chosen based on the specific application requirements. For instance, MIM WGs offer extremely tight mode confinement, making them suitable for integrated photonic circuits [16,17,18,19]. IMI WGs, on the other hand, are less confined but suffer from lower propagation losses, making them advantageous for practicalities requiring longer propagation lengths [20].

An MIM WG is one of the most studied plasmonic WG structures due to its ability to confine light at nanoscale dimensions [21,22,23]. Several novel structures such as filters [24,25,26,27,28], modulators [29,30,31,32], sensors [18,33,34,35], splitters [36,37,38,39,40], a demultiplexer [41,42,43,44], and switches [45,46,47] have recently been proposed for their eye-catching practicalities. A typical MIM WG comprises a thin dielectric layer sandwiched between two metal layers. The high contrast in the refractive index (RI) between the metal and the dielectric material results in strong confinement of the EM fields within the dielectric layer. In an MIM WG, the dielectric layer is typically in the order of tens of nanometers thick, which allows the supported plasmonic modes to be confined to dimensions much smaller than the wavelength of light in free space [48]. The EM field in the MIM WG decays exponentially into both the metal and dielectric regions, with much of the field confined to the dielectric layer. This confinement is a result of the SPR conditions at the metal–dielectric interfaces, where the collective oscillation of free electrons in the metal interacts with the EM field [49].

**Figure 1:**
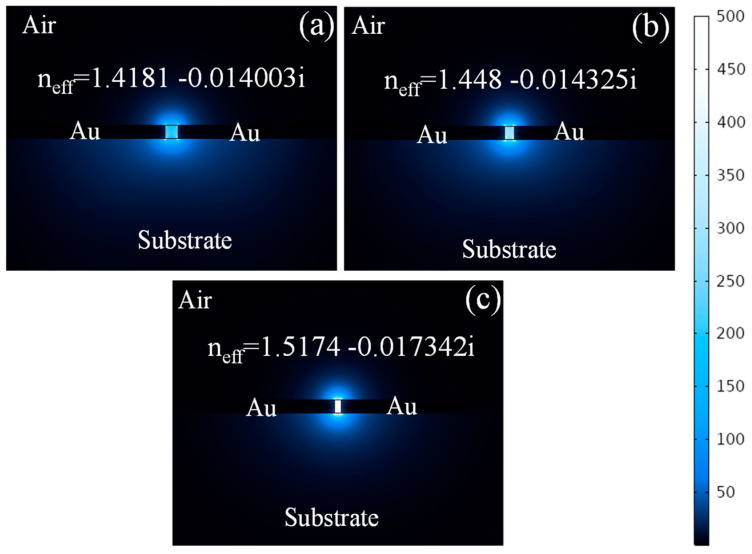
Norm. E-field pattern in the MIM WG for the nanoslot of (**a**) 100 nm, (**b**) 75 nm, and (**c**) 50 nm.

The dispersion relation of MIM WGs is non-trivial and shows that the effective index of the guided mode can be significantly higher than the RI of the dielectric material [13]. For instance, the E-field distribution in the MIM WG for the operational wavelength of 1550 nm is shown in Figure 1. As the width of the nanoslot decreases, the effective mode index increases, leading to stronger field confinement and higher propagation losses, as shown in Figure 1a–c. The E-field distribution patterns are obtained from the COMSOL Multiphysics software 6.0. The high effective index also means that the phase velocity of the guided mode is reduced, which is a critical factor for implementations in slowing light [50] and enhancing light–matter interactions [51].

A significant challenge in MIM WGs is the inherent loss due to the absorption in the metal layers. Metals such as gold (Au) and silver (Ag), commonly used in plasmonics, exhibit considerable ohmic losses at optical frequencies [52]. These losses manifest as a decay in the amplitude of the guided mode as it propagates, limiting the practical length of MIM WGs. Various strategies have been proposed to mitigate these losses, including the use of alternative materials like transparent conductive oxides or hybrid structures that combine plasmonic and dielectric WGs. Despite the high losses, MIM WGs are highly attractive for a variety of functions. Their ability to confine light to very small volumes make them ideal for enhancing nonlinear optical effects, which are typically weak in bulk materials. This enhancement is useful in functions such as all-optical switching, modulators, and frequency converters [29,46,53]. Moreover, the strong confinement in MIM WGs can substantially enhance the sensitivity (S) of plasmonic sensors, enabling the detection of minute changes in the RI of the surrounding environment, which is valuable in biosensing and environmental monitoring [16,54,55].

**Figure 2:**
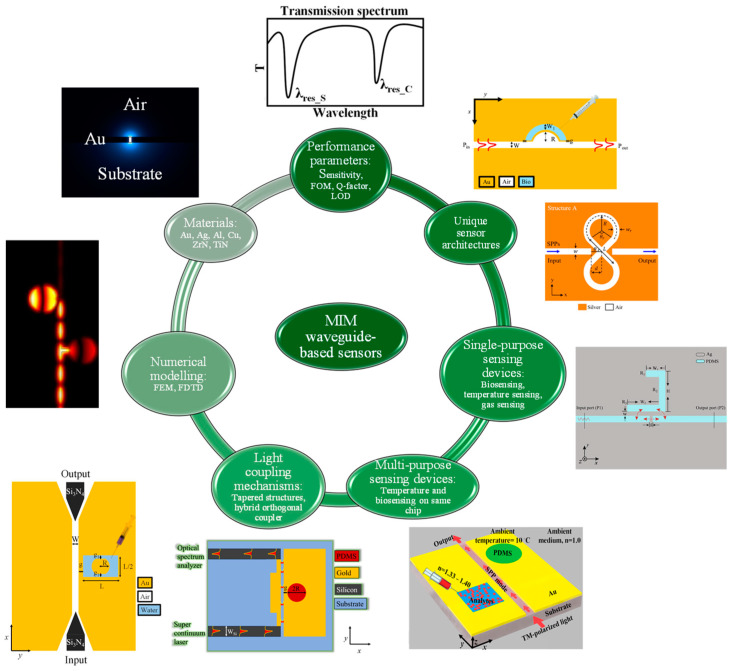
Graphical illustration of the topics discussed in this paper, which include performance parameters, material platforms, numerical methods, sensing devices, and light-coupling mechanisms.

The paper is categorized as follows: Section 2 delves into the performance parameters of plasmonic sensors established on MIM WGs, including sensitivity (S), figure of merit (FOM), Q-factor, and limit of detection (LOD). Section 3 is dedicated to the numerical methods utilized for the analysis of plasmonic devices, with an in-depth discussion on two primary techniques: the finite element method (FEM) and the finite difference time domain (FDTD) method. Section 4 addresses both conventional and emerging plasmonic materials used in the design of MIM-WG-based sensors. Section 5 presents a critical analysis of the sensing performance of MIM-WG-based plasmonic sensors compared to conventional integrated photonic sensors. Section 6 explores MIM-WG-based plasmonic sensors, categorized into two subsections based on their application to single- or multi-parametric sensing. Section 7 delves into the light coupling mechanisms for MIM WGs, including discussions on tapered WG structures and hybrid orthogonal couplers. Section 8 presents the challenges and obstacles in the development of MIM-WG-based plasmonic sensing devices. The paper concludes with a concise summary in Section 9. Figure 2 provides the outline of the review paper.

## 2. Performance Parameters of MIM-WG-Based Plasmonic Sensing Devices

S is a crucial parameter in the performance of MIM-WG-based plasmonic sensors as it determines the sensor’s ability to identify minute variations in the RI of the surrounding medium [56,57]. In the context of plasmonic sensors, S is often quantified as the shift in the resonance wavelength (λ_res_) per unit change in the RI (usually expressed in nm/RIU, where RIU stands for refractive index unit) and expressed as [13]

(1) $$Sensitivity\;\left(\frac{nm}{RIU}\right)=\frac{∆\lambda }{∆n}$$

The design of the MIM WG plays a significant role in optimizing S. Parameters such as the thickness of the insulating layer, the choice of metal (commonly gold or silver), and the geometrical configuration of the WG impact the field confinement and, consequently, the S. For instance, reducing the thickness of the insulator can increase the field confinement, thereby enhancing S [18,33]. The use of graphene and other two-dimensional materials can enhance the plasmonic response due to their high surface area and strong light–matter interaction [58,59]. Moreover, integrating MIM WGs with nanofluidic channels allows precise control over the analyte’s position relative to the sensor, optimizing the sensing conditions and further enhancing S.

The FOM is another critical metric used to evaluate the performance of MIM-WG-based plasmonic sensors [60,61]. The FOM is typically expressed as the ratio of S to the full width at half maximum (FWHM) of the resonance peak and expressed as [13]

(2) $$FOM=\frac{Sensitivity}{FWHM}$$

This ratio provides a measure of the sensor’s ability to distinguish between small changes in the RI while maintaining a sharp resonance peak. A higher FOM indicates a more effective sensor, capable of detecting subtle variations in the analyte concentration with high resolution. The FOM is influenced by both the intrinsic properties of the materials used and the design of the MIM WG. Materials with low optical losses, such as noble metals (gold and silver), are preferred because they contribute to narrower resonance peaks, thereby enhancing the FOM. Additionally, the WG geometry, including factors like the aspect ratio and the presence of structural modifications (e.g., gratings or resonators), can be engineered to achieve a high FOM [62,63]. For example, introducing defects or resonant cavities within the MIM structure can lead to sharper resonance peaks, thus improving the FOM [64]. Optimizing the FOM also involves a trade-off between S and the Q-factor of the resonance [65]. While increasing the field confinement can boost S, it may also broaden the resonance peak due to increased absorption losses. Therefore, achieving a high FOM requires a careful balance between these factors [66].

For Lorentzian line shapes, which are associated with simple resonant systems, the FOM tends to be moderate due to the broader and symmetric nature of the resonance peaks, reflecting moderate S and resolution [13,67]. In contrast, the Fano resonance, characterized by its asymmetric line shape with sharp peaks and dips, exhibits a significantly higher FOM [18]. This is because the Fano resonance’s narrow FWHM, combined with its pronounced S to environmental changes, allows for a more precise detection of shifts in λ_res_. Consequently, MIM WG sensors utilizing Fano resonance demonstrate superior performance, providing enhanced S and higher resolution compared to those based on Lorentzian line shapes, making them more effective for sensing minute changes in analyte concentrations [68,69].

The limit of detection (LOD) is another critical parameter that defines the sensor’s ability to detect the smallest possible quantity of an analyte [70]. MIM WG structures leverage the confinement and propagation of SPPs at the metal–insulator interfaces, improving the interaction between the analyte and the sensor surface. This pattern allows for the detection of minute variations in the RI near the sensor surface, thereby enabling high S [71]. For plasmonic sensors, the formula can be expressed as

LOD = 3σ/S;(3)

where σ is the standard deviation of the noise in the sensor’s signal (baseline noise). S is the sensitivity of the plasmonic sensor, typically defined as the shift in the plasmon λ_res_ or intensity per unit change in the analyte concentration. The factor of 3 is used to ensure that the detected signal is distinguishable from the noise with a high confidence level (usually 99%). Therefore, the LOD for a plasmonic sensor quantifies the minimum detectable change in the analyte concentration that produces a signal three times greater than the standard deviation of the noise. This formula helps determine the sensor’s ability to detect low concentrations of analytes accurately.

The Q-factor, or quality factor, of plasmonic sensors is a crucial parameter that determines the efficiency and performance of these devices in various sensing applications [72]. It measures the ratio of the energy stored in the plasmonic modes to the energy dissipated per cycle, with a higher Q-factor indicating lower energy losses and sharper resonance peaks. The Q-factor can be stated as

Q-factor = λ_res_/FWHM; (4)

where λ_res_ is the resonance wavelength and FWHM is the full width at half maximum of the spectral line. This sharpness enhances the sensor’s ability to sense minute variations in the RI or the presence of specific analytes with high S and precision. The Q-factor is influenced by factors such as the materials used (e.g., metals like Au and Ag), the geometric configuration of the sensor, and the coupling efficiency (CE) of the plasmonic modes [73,74]. Optimizing these factors can lead to improved Q-factors, thereby enhancing the resolution and reliability of plasmonic sensors in applications ranging from biochemical detection to environmental monitoring and medical diagnostics.

## 3. Numerical Methods to Simulate Photonic Devices

Numerical tools for simulating photonic devices are fundamental for precision in the design and optimization of complex optical structures. Among these, the finite element method (FEM) and finite difference time domain (FDTD) method stand out for their ability to capture intricate EM interactions. The FEM excels in modeling devices with complex, inhomogeneous materials by discretizing the structure into finite elements, allowing detailed control over boundary conditions and material properties. The FDTD method, in contrast, is particularly advantageous for simulating time-dependent wave behavior across various geometries, making it ideal for photonic crystals, waveguides, and metamaterials, where intricate light–matter interactions are essential. Together, these powerful tools empower researchers to push the limits of photonic device performance, streamline fabrication, and drive innovation in fields like optical communication, sensing, and quantum computing.

The finite element method (FEM): It is an overwhelming numerical technique extensively used for simulating photonic devices. The FEM is particularly well-suited for problems involving complex geometries and inhomogeneous materials, which are common in photonics. The method involves discretizing the problem domain into small, non-overlapping elements, typically triangles or tetrahedra in two or three dimensions, respectively [75]. These elements form a mesh that approximates the physical structure of the photonic device. The core of the FEM lies in solving Maxwell’s equations, which govern the behavior of EM fields in photonic devices. By converting these differential equations into a set of algebraic equations, the FEM makes the problem tractable for computer simulation. This conversion is achieved through a process known as variational formulation or the method of weighted residuals, where the continuous partial differential equations are transformed into a system of discrete equations [76].

The FEM is particularly advantageous for photonic devices due to its flexibility in handling complex boundaries and interfaces between different materials. For instance, in simulating WGs, photonic crystals, plasmonic devices, or metamaterials, the ability to accurately model the geometry and material properties is crucial for predicting device performance. The FEM can also accommodate anisotropic materials and non-linear optical effects, further enhancing its applicability in advanced photonic designs. Moreover, the FEM can be efficiently implemented to solve both time-independent (frequency domain) and time-dependent (time domain) problems. In the frequency domain, the FEM can be used to determine the eigenmodes of photonic structures, such as the resonant modes of cavities or the guided modes of WGs [77]. In the time domain, the FEM can simulate the transient response of photonic devices, capturing the dynamics of light–matter interaction over time [78].

The accuracy and convergence of the FEM depend on the mesh quality and the order of the polynomial basis functions used to approximate the solution within each element. Higher-order basis functions can provide more accurate results but at the cost of increased computational complexity. Adaptive mesh refinement techniques are often employed to enhance accuracy in regions with high field gradients while keeping the computational load manageable [79].

The finite difference time domain (FDTD) method: The FDTD method is another prominent numerical technique extensively used for simulating photonic devices. The FDTD method operates by discretizing both space and time, enabling the direct solution of Maxwell’s curl equations. This method is particularly well-suited for time-dependent problems and is highly effective for modeling the propagation of EM waves through various photonic structures [80]. The FDTD method divides the simulation domain into a grid of Yee cells, named after Kane Yee, who introduced this scheme [81]. Each cell in the grid holds the electric and magnetic field components, which are updated alternately in time using finite difference approximations of Maxwell’s equations. This leapfrog updating process ensures that the electric and magnetic fields remain coupled and evolve accurately over time [82].

One of the main advantages of the FDTD method is its ability to model broadband responses in a single simulation run. Since the FDTD method is a time-domain method, it inherently captures the full spectrum of the EM response as the fields propagate through the photonic device. This feature is particularly useful for analyzing devices that interact with a wide range of wavelengths, such as photonic crystals, metamaterials, and plasmonic structures. The FDTD method is also highly adaptable to various boundary conditions, including Perfectly Matched Layers (PML) and Mur’s absorbing boundaries, which are essential for minimizing reflections from the edges of the simulation domain [83]. This adaptability allows the FDTD method to simulate open-region problems effectively, ensuring that the results are not contaminated by artificial boundary reflections [84].

In terms of implementation, the FDTD method is straightforward and scales well with computational resources. The method is inherently parallelizable, making it suitable for high-performance computing environments. This scalability is crucial for handling large-scale simulations required in the design and optimization of complex photonic devices [85].

However, the FDTD method does have some limitations. The method requires fine spatial and temporal discretization to accurately resolve high-frequency components and small features, leading to significant computational and memory demands [86]. Additionally, the FDTD method can face challenges when dealing with highly dispersive or anisotropic materials, although advanced techniques and modifications, such as the auxiliary differential equation (ADE) and multi-resolution time-domain (MRTD) methods, have been developed to address these issues. Despite these challenges, the FDTD method remains a highly valuable tool in the simulation of photonic devices. Its ability to provide detailed temporal and spatial field patterns makes it indispensable for understanding the dynamic behavior of photonic structures, facilitating the development of innovative devices with tailored EM properties [87].

## 4. Conventional and Emerging Material Platforms

Plasmonic devices leverage the unique interaction between light and free electrons in metals to manipulate light at the nanoscale [88]. This interaction is primarily facilitated by the phenomenon of SPR, which occurs when the collective oscillations of free electrons on a metal surface resonate with the incident light. The metals most commonly used for plasmonic applications are gold (Au), silver (Ag), and aluminum (Al), each offering distinct advantages and challenges.

Au is arguably the most extensively used metal in plasmonic devices due to its exceptional chemical stability and well-understood optical properties [89]. It exhibits strong SPR in the visible to near-infrared region, making it ideal for bioimaging, sensing, and photothermal therapy applications [90]. Au nanoparticles have been widely researched for their ability to enhance EM fields at their surfaces, leading to significant advancements in surface-enhanced Raman spectroscopy (SERS) and other sensing technologies. Moreover, Au’s biocompatibility makes it suitable for in vivo applications, including targeted drug delivery and diagnostic imaging. One of the main advantages of Au is its oxidation resistance, ensuring the long-term stability and reliability of plasmonic devices [91]. However, Au also has limitations. Its relatively high cost and the need for precise fabrication techniques can be prohibitive for large-scale applications. Additionally, Au’s plasmonic performance, while excellent in the visible spectrum, diminishes in the ultraviolet region, limiting its use in certain applications that require shorter wavelengths [92].

Ag possesses the highest plasmonic efficiency among all metals, with the strongest SPR in the visible and near-ultraviolet regions [93]. This makes Ag an attractive choice for applications such as biosensing, SERS, and photonic devices [94,95]. The superior optical properties of Ag stem from its low intrinsic losses and high electrical conductivity, enabling more effective confinement and enhancement of EM fields. Despite its advantages, Ag’s practical use in plasmonic devices is often hindered by its susceptibility to oxidation and tarnishing when exposed to air or certain environments [96]. This degradation can significantly affect the performance and durability of Ag-based plasmonic devices. Researchers have developed various strategies to mitigate this issue, including protective coatings and alloying with other metals, but these solutions can add complexity and cost to the fabrication process.

Al is gaining attention in the field of plasmonics, especially for applications in the ultraviolet (UV) region, where gold and silver are less effective [97,98]. Its plasmonic resonance can be tuned across a broad spectrum, including deep UV, making it valuable for applications in UV lithography, UV sensors, and photocatalysis. Additionally, Al is abundant and cost-effective, offering a significant advantage for scalable and economically viable plasmonic devices [99]. However, Al also faces challenges, primarily related to its relatively high losses in the visible spectrum and its tendency to form a native oxide layer. This oxide layer can affect the plasmonic properties, though it can also serve as a natural protective barrier against further oxidation. Advances in nanofabrication techniques have enabled the precise control of Al’s properties, enhancing its potential for high-performance plasmonic applications [100].

**Figure 3:**
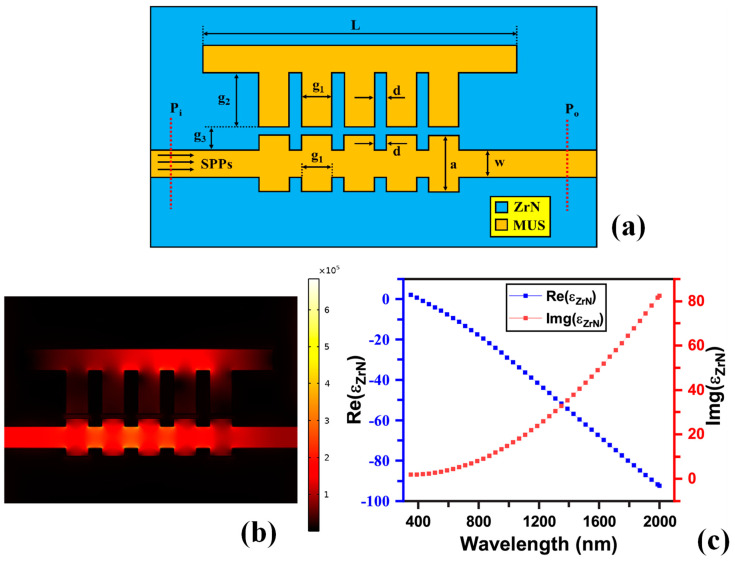
(**a**) Graphical illustration of the sensor [110], (**b**) E-field pattern at the resonant wavelength [110], and (**c**) real and imaginary parts of permittivity of ZrN in the visible and near-IR spectrum [110].

Beyond these primary metals, researchers are exploring other materials and alloys to enhance and expand the capabilities of plasmonic devices [101]. Copper (Cu), for instance, offers good plasmonic properties and is more cost-effective than Au and Ag, although it is also prone to oxidation [102,103,104]. Transition metal nitrides, such as titanium nitride (TiN) [105,106,107] and zirconium nitride (ZrN) [108,109,110], are emerging as promising alternatives due to their stability and tunable optical properties, extending the operational range of plasmonic devices into harsher environments and higher temperatures. A novel comb-shaped plasmonic RI sensor employing a ZrN–insulator–ZrN configuration was introduced by Rakib et al., as shown in Figure 3a [110]. The E-field pattern in the device at a resonant wavelength is shown in Figure 3b. Figure 3c displays the real and imaginary parts of the permittivities, demonstrating that ZrN can support SPPs at the ZrN–MUS interface. ZrN, which is compatible with universal Complementary Metal Oxide Semiconductor (CMOS) processes, exhibits tunable optical properties. The sensor achieved a maximum S (1445.46 nm/RIU), FOM of 140.96, and sensing resolution of 6.91 × 10^−7^ RIU^−1^. Additionally, incorporating ZrN imparted several advantages to the sensor: enhanced hardness, thermal stability at high temperatures, improved resistance to corrosion and abrasion, and lower electrical resistivity. These properties are lacking in traditional plasmonic metals, limiting the practical application of plasmonic devices. Consequently, the proposed model outperformed the conventional noble-metal-based MIM structure, offering the potential for highly efficient, robust, and durable nanoscale sensing devices. This advancement could bridge the gap between nanoelectronics and plasmonics [110].

Moreover, hybrid materials and structures, such as metal–dielectric composites and metal–organic frameworks [111,112], are being investigated to combine the benefits of different materials and overcome individual limitations. These innovations are pushing the boundaries of what is achievable with plasmonic devices, opening new avenues for advanced photonic and optoelectronic applications. While Au, Ag, and Al remain the cornerstone metals for plasmonic devices, ongoing research and development are continually broadening the range of materials and improving these sophisticated technologies’ performance, durability, and scalability.

## 5. MIM-WG-Based Plasmonic Sensors Versus Conventional Integrated Photonic Sensors

Typical integrated photonic sensors, which typically rely on dielectric WGs, are inherently less sensitive than MIM-WG-based sensors due to several fundamental differences in their operating principles and physical characteristics. The S of a sensor is largely dictated by how effectively it can confine and interact with the optical field in the presence of the analyte [113,114,115]. In dielectric WG sensors, the optical mode is predominantly confined within the core of the WG, which limits the interaction with the external environment where the analyte is present. The evanescent field, which extends into the surrounding medium and is responsible for sensing, is relatively weak and decays rapidly [116]. This results in a lower overlap between the optical field and the analyte, reducing the overall S of the sensor [71,117]. Apart from ridge-WG-based sensors, several other attractive WG configurations are widely used to enhance the S of the device [118,119,120,121,122].

In contrast, MIM-WG-based sensors exploit the unique properties of SPPs, which are EM waves that transmit along the interface between a metal and an insulator. These SPPs are highly confined to the metal surface, with an electromagnetic field that extends extensively into the surrounding medium [123]. This strong field confinement and enhanced interaction with the environment lead to a much higher S to changes in the RI or the presence of molecular species. The enhanced field–matter interaction is a direct consequence of the plasmonic effect, where the free electrons in the metal resonate with the incident light, creating an intensified electromagnetic field at the metal–insulator interface. This heightened S makes MIM-WG-based sensors particularly effective in detecting low-concentration analytes and minute changes in the local environment [17]. The fundamental attributes of MIM-WG-based sensors are presented in Table 1.

Furthermore, MIM-WG-based sensors offer greater design flexibility in tuning their optical properties. By carefully selecting the materials and adjusting the structural dimensions of the WG, such as the thickness of the metal and insulator layers, it is possible to manipulate the propagation characteristics of the SPPs. This tunability allows for the optimization of sensor performance for specific applications, enhancing S and selectivity [124]. In contrast, dielectric WG sensors have more limited design parameters, as their optical properties are primarily governed by the refractive indices of the core and cladding materials, and the WG dimensions [125].

Another critical factor contributing to the superior S of MIM-WG-based sensors is their ability to operate at nanoscale dimensions. The plasmonic modes in MIM structures can be confined to volumes much smaller than the wavelength of light, facilitating the development of ultra-compact sensors [126]. This nanoscale confinement not only enhances S but also enables the integration of multiple sensing elements within a small footprint, increasing the potential for multiplexed sensing and high-throughput analysis. Standard dielectric WG sensors, while also capable of miniaturization, do not achieve the same level of field confinement and thus cannot match the S levels of MIM-based sensors in such compact formats [127].

## 6. MIM-WG-Based Sensing Devices

In this section, MIM-WG-based sensing devices with different and attractive cavity shapes are discussed and divided the sections into two categories: single-parametric sensing devices and multi-parametric sensing devices. Table 2 presents the recently proposed, highly attractive MIM-WG-based plasmonic devices for diverse sensing applications.

### 6.1. Single-Parametric Sensing Devices Established on MIM WG

Researchers propose various cavity shapes for MIM-WG-based sensors to explore and optimize their optical properties and sensing capabilities. Different cavity shapes can significantly influence the EM field pattern, resonance characteristics, and transmission spectra of the sensors. By modifying the geometry of the cavities, researchers can tailor the spectral response, including the λ_res_ and line widths, which are crucial for sensing applications. Moreover, varying cavity shapes can enhance the S of the device to changes in the RI of the surrounding medium, thereby improving its performance as a plasmonic sensor. Therefore, the investigation and proposal of different cavity shapes play a pivotal role in advancing the capabilities and applications of MIM-WG-based sensors in optical sensing technologies. In this section, the MIM-WG-based plasmonic sensors with different cavity shapes are discussed.

#### 6.1.1. Biosensing

MIM-WG-based plasmonic sensors are employed for several interesting applications [51,128,129,130]. A RI sensor is significant due to its pivotal role in various scientific and industrial applications, particularly in the fields of chemistry, biology, and environmental monitoring [58,131]. These sensors operate by measuring the RI of a medium, which changes in response to variations in composition, concentration, or other properties of the substance being analyzed [54]. This capability makes RI sensors invaluable for detecting minute changes in chemical concentrations, monitoring biomolecular interactions, and ensuring quality control in manufacturing processes. Their high S, real-time analysis, and non-invasive nature allow for precise and efficient monitoring, contributing to advancements in medical diagnostics, pharmaceuticals, food safety, and environmental protection [128].

**Figure 4:**
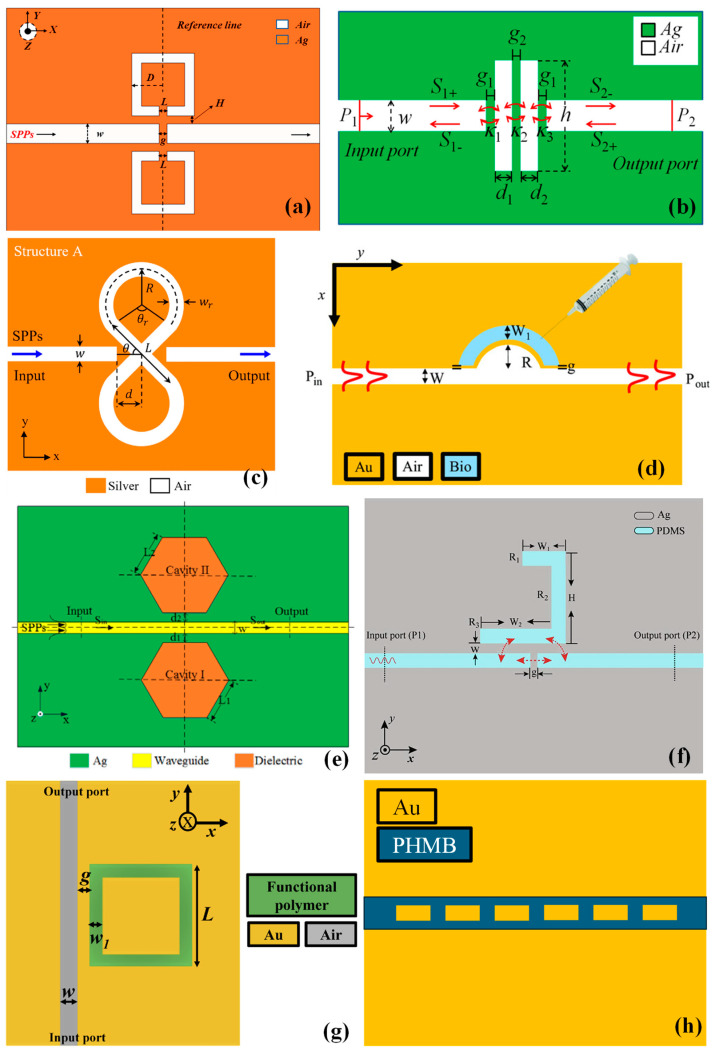
Plasmonic devices based on MIM WG for (**a**) biosensing [67], (**b**) biosensing [68], (**c**) biosensing [72] (**d**) biosensing [21], (**e**) temperature sensing [133], (**f**) gas sensing [22], and (**g**,**h**) gas sensing [34].

Chen et al. reported a non-through MIM WG capable of exciting five-fold Fano resonances [67]. These resonances originated from the interaction between modes excited by the square split-ring resonator (SSRR) and the bus WG, as shown in Figure 4a. Through a detailed analysis using the FEM of the transmission characteristics and magnetic field strength, it was determined that the Fano λ_res_ and transmittance could be independently tuned by regulating the structural dimensions of the SSRR. Upon optimizing these structural dimensions, the structure achieved optimal RI-sensing S and an FOM of 1290.2 nm/RIU and 3.6 × 10^4^, respectively. Furthermore, the annular cavity of the MIM WG structure can be loaded with a biomass solution to function as a biosensor. Consequently, this configuration is suitable for optical RI sensing in biological, micro-, and nano-fields.

An RI sensor utilizing MIM WGs coupled with double rectangular cavities was proposed and numerically analyzed using the FEM by Zhang et al., as shown in Figure 4b [68]. The transmission properties and RI S of different sensor configurations were thoroughly examined. The sensor’s transmission spectra displayed an asymmetric Fano resonance line shape, resulting from the interference between a broad resonance mode in one rectangular cavity and a narrow mode in the other. The impact of various structural parameters on the Fano resonance and the system’s RI S was studied. The proposed plasmonic RI sensor achieved a maximum S of 596 nm/RIU [68].

In another study, an 8-shaped resonator coupled to MIM WGs was utilized to design plasmonic filters and sensors, as depicted in Figure 4c [72]. The resonator supported two resonance modes, leading to peaks in the structure’s transmission spectrum. A quality (Q) factor of 247.4, which could increase to 270 at a wavelength of 1187.5 nm, was witnessed. By incorporating vertical and horizontal metal blades within the resonator, two tunable single-mode plasmonic filters were achieved at the first and second resonance modes, respectively. The effects of structural parameters on the transmission spectrum were analyzed using the FDTD method. The results indicate that the plasmonic structure was suitable for biosensing applications, such as detecting basal cancer cells, with an S of 1200 nm/RIU.

Furthermore, the study included a numerical analysis of a plasmonic sensor based on an MIM WG for RI-sensing applications [21]. This sensor configuration featured an MIM bus waveguide with a semicircular side-coupled to a semicircular cavity, as depicted in Figure 4d. Notably, this design achieved a transmission dip characterized by a higher extinction ratio (ER) compared to the standard MIM waveguide side-coupled to a semicircular cavity. To address the challenge of light coupling to the MIM plasmonic waveguide, mode converters were seamlessly integrated into the system. The device demonstrated an impressive S of approximately 941.33 nm/RIU, positioning it as a highly promising candidate for point-of-care testing applications. Such applications typically involve the use of portable or handheld diagnostic devices to swiftly analyze samples of bodily fluids like blood or urine. This capability is crucial for diagnosing a wide array of conditions ranging from infections to chronic diseases such as diabetes or heart disease [21].

#### 6.1.2. Temperature Sensing

Temperature sensing is another important application that is widely studied for MIM-WG-based plasmonic sensors [123,132,133,134,135]. Plasmonic temperature sensors offer high S, rapid response times, and the capability for real-time monitoring. They are especially useful in applications requiring localized temperature measurements at the micro- or nanoscale, such as in microfluidics, biological research, and electronic device monitoring. In microfluidics, for example, plasmonic temperature sensors can monitor the temperature of small volumes of fluids, which is crucial for various biochemical reactions. In biological research, they can measure the temperature within cells or tissues, providing insights into cellular processes and metabolic activities.

Polydimethylsiloxane (PDMS) polymer is increasingly being utilized in MIM-WG-based sensors for temperature sensing due to its unique optical and material properties [123]. PDMS is a flexible, transparent, and biocompatible silicone-based polymer that exhibits a significant thermo-optic effect, meaning its RI changes with temperature [136]. This property makes PDMS an ideal candidate for integration into photonic sensor designs, where the S to temperature variations is critical. In a typical configuration, a WG or resonator structure is embedded in or coated with PDMS, allowing the device to detect temperature changes through shifts in optical characteristics such as λ_res_ or transmission intensity [137]. The material’s high thermal expansion coefficient and stability over a wide temperature range further enhance the S and reliability of the sensors. Additionally, PDMS’s ease of fabrication and ability to conform to various shapes and surfaces enable the development of versatile and cost-effective temperature sensors [138]. These sensors find applications in numerous fields, including biomedical monitoring, environmental sensing, and industrial process control, where accurate and responsive temperature measurement is essential [132].

Ethanol is another effective temperature-sensing material due to its well-defined thermo-optic properties, which include a significant change in the RI with temperature [139,140]. When integrated into plasmonic sensor systems, ethanol’s RI variation with temperature can be precisely monitored using optical methods such as interferometry, fiber Bragg gratings, or surface plasmon resonance [141,142]. This capability allows for accurate temperature measurements over a wide range of environmental conditions. Furthermore, ethanol’s chemical stability and compatibility with various photonic materials make it a versatile choice for diverse applications, including environmental monitoring, industrial processes, and biomedical diagnostics [143]. The S and reliability of ethanol-based plasmonic temperature sensors are enhanced by their rapid response to temperature changes, facilitating real-time monitoring and control in critical applications [144].

A novel plasmonic temperature-sensing structure was proposed by Xie et al., featuring an MIM WG with dual, symmetric, coupled hexagonal cavities, as illustrated in Figure 4e [133]. Simulation results obtained using the FDTD method demonstrated that the full width at half maximum (FWHM) of the resonance dip can be narrowed by decreasing the coupling distance between the MIM WG and the cavities. Additionally, an optimal ER can be achieved at a specific position. The temperature-sensing characteristics were investigated based on the relationship between the RI of the dielectric material (ethanol) and the ambient temperature. The subsequent temperature S, determined through spectral interrogation, was found to be approximately 0.45 nm/°C, with an FWHM of 34 nm and an ER of about 19 dB at a coupling distance of 23 nm. This research contributes to the design of nanoscale optical sensors with high temperature S and high sensing resolution.

In another instance, a compact temperature sensor established on Fano resonance was proposed by Zhu et al. [123]. The sensor integrates two MIM WGs with a side-coupled semi-square ring resonator sealed with PDMS, as shown in Figure 4f. Using FEM simulations, the transmission characteristics and optical properties of the plasmonic structure were comprehensively analyzed, achieving excellent performance. The results indicated distinct transmission–reflection profiles in the spectra, with Fano resonance curves showing sensitive responses to variations in structural parameters and ambient temperature. Leveraging PDMS’s high thermo-optic coefficient, the sensor demonstrated exceptional S, reaching up to −4 nm/°C. 

While the thermo-optical properties of PDMS and ethanol provide significant advantages for temperature-sensing applications, particularly due to their sensitivity and ease of fabrication, it is essential to consider whether these properties remain stable in varied, real-world conditions beyond the controlled environment of a laboratory. Factors such as long-term material stability and potential degradation are critical, as both PDMS and ethanol may be susceptible to environmental stressors like UV exposure, humidity, or fluctuating temperatures over time. Such conditions could lead to changes in optical clarity, the refractive index, or even physical deformation, potentially compromising the sensor’s accuracy and reliability. Additionally, ethanol’s volatility poses challenges, as evaporation rates can vary with ambient temperature and pressure, impacting the consistency of the sensor’s readings [145]. A thorough examination of these factors would be crucial for validating the suitability of PDMS- and ethanol-based sensors in complex, diverse environments, where maintaining calibration and sensor performance is necessary for long-term application success.

#### 6.1.3. Gas Sensing

Gas sensors play a pivotal role in modern society, offering indispensable functionalities across various domains [116,146]. These sensors are crucial for monitoring and detecting the presence of gases in diverse environments, ranging from industrial settings to everyday applications in homes and vehicles. By continuously analyzing the composition of the air or specific gases, they ensure safety, efficiency, and environmental sustainability. In industrial contexts, gas sensors are essential for maintaining workplace safety by detecting hazardous gases such as carbon monoxide, hydrogen sulfide, and volatile organic compounds (VOCs). Early detection through these sensors can prevent accidents, protect workers’ health, and mitigate potential environmental damage. Moreover, in manufacturing processes, gas sensors contribute to quality control and efficiency by monitoring gas levels critical to production and ensuring compliance with regulatory standards [147].

Beyond safety, gas sensors are integral to environmental monitoring and pollution control efforts. They facilitate real-time air quality measurements in urban areas, helping governments and organizations to make informed decisions to mitigate pollution and safeguard public health. Additionally, in agricultural settings, these sensors aid in optimizing conditions for plant growth by monitoring gases such as carbon dioxide and methane in greenhouses. The advancements in gas sensor technology continue to expand their applications, with innovations like miniaturization enabling integration into wearable devices and smartphones for personal air quality monitoring. Thus, their continuous development and deployment are crucial for a safer, healthier, and more efficient future [148].

The study presented by Khonina et al. explored a novel CO_2_ gas sensor design using the FEM. The sensor integrated a plasmonic MIM WG coupled to a square ring cavity filled with polyhexamethylene biguanide (PHMB) polymer, which is a functional polymer and exhibits a linear response to CO_2_ concentration, making it suitable for CO_2_ sensing applications, as shown in Figure 4g [22]. Employing PHMB in the MIM WG achieved an S of 135.95 pm/ppm, surpassing Si photonics-based optical sensors. This S was approximately seven times greater than the previously reported values. These findings hold promise for advancing sensors capable of detecting toxic gases using various functional materials.

In another instance, Butt et al. proposed two novel configurations of modified plasmonic Bragg grating structures incorporating a functional polymer for applications in filtering and gas sensing, as shown in Figure 4h [34]. The symmetric configuration of the transformed Bragg grating was ideal for developing a broad-bandwidth bandstop filter. Tailoring the device’s geometric dimensions allows for optimizing key filter parameters such as the ER, stopband characteristics, and bandwidth. The asymmetric configuration of the transformed Bragg grating involved displacing periodically arranged Au nano-blocks within the MIM WG away from the symmetry axis. This modification introduced an extra narrowband resonance dip in the transmission spectrum, alongside the broad Bragg reflection. The spectral characteristics of these proposed devices were analyzed using the FEM. The S of the CO_2_ gas sensing device was approximately 226 pm/ppm over a concentration range of 215 ppm to 434 ppm, significantly surpassing many existing sensor designs. 

### 6.2. Multi-Parametric Sensing Devices Established on MIM WG

Multi-parametric sensing capabilities of plasmonic sensors are highly significant because they enable the simultaneous detection and analysis of multiple environmental parameters with high precision and speed. This multifaceted approach enhances the sensor’s ability to provide comprehensive and detailed information about complex systems, making it invaluable in applications such as environmental monitoring, healthcare diagnostics, and industrial process control [89,132]. The integrated sensing capability reduces the need for multiple sensors, leading to cost savings, a simplified system design, and more robust and reliable data collection, ultimately driving advancements in technology and improving decision-making across various fields.

**Figure 5:**
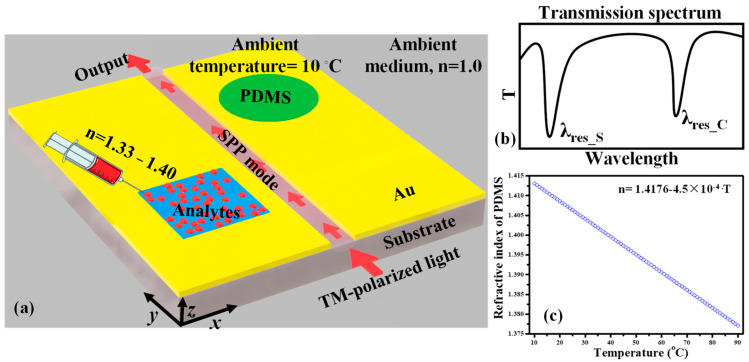
(**a**) Graphical illustration of a plasmonic sensing device for concurrent detection of biological samples and temperature [149], (**b**) transmission spectrum of the device showing two independent resonant dips [149], and (**c**) deviation in the RI of the PDMS material set against the ambient temperature [149].

The research devoted to MIM-WG-based sensors is mainly focused on single-parametric sensing; however, little attention has been paid to the simultaneous detection of multiple parameters. For instance, Kazanskiy et al. anticipated a multi-parametric sensing device established on an MIM WG for both biosensing and temperature-sensing applications [149]. The device featured two cavities—a square cavity (SC) and a circular cavity (CC)—side-coupled to an MIM bus WG, as shown in Figure 5a. For biosensing, analytes were introduced into the SC, while a thermo-optic polymer was placed in the CC, inducing a λ_res_ shift in response to ambient temperature variations. These sensing processes operated independently, with each cavity producing a distinct resonance dip in the transmission spectrum (Figure 5b), ensuring a clear, unobstructed analysis. This straightforward configuration on a single chip offered impressive S, achieving 700 nm/RIU for biosensing and −0.35 nm/°C for temperature sensing. The relationship between the RI of PDMS and the ambient temperature is presented in Figure 5c. The FOM, defined as the ratio of device S to the width of the resonance dip, is approximately 21.9 for biosensing and 0.008 for temperature sensing. This sensor design holds significant potential: (i) for testing biological analytes in regulated temperature environments and (ii) for mitigating the impact of ambient temperature variations on refractometric measurements in real-time applications [149].

In another instance, Zhang et al. conducted a detailed analysis of a multifunctional optical sensor capable of simultaneously detecting pressure and temperature, utilizing an MIM WG structure with two T-shaped cavities [132]. The simulations indicated that pressure and temperature can be independently measured by these cavities at distinct Fano λ_res_ values. For pressure sensing, the upper T-shaped cavity exhibited a linear λ_res_ shift as pressure increased due to slight deformation, achieving a maximum S of 12.48 nm/MPa. Beyond a certain pressure threshold, the pressure–resonance relationship became quadratic. The lower T-shaped cavity incorporated solid PDMS as a thermally sensitive material, which prevented material overflow due to structural micro-vibrations and ensures high temperature S with a coefficient of 0.36 nm/°C. By adjusting the selection of Fano resonances, the sensor ensured independent and interference-free measurements of pressure and temperature. This innovative design offered extensive application potential for environments requiring multiparameter monitoring, enhancing the precision and reliability of complex sensing tasks [132].

**Table 2:** Recently proposed MIM-WG-based sensors and their sensing characteristics.

Cavity Shape	Materials	Applications	S	FOM	Q-Factor	Ref.
Bearing circular ring cavity	Ag	Biosensing	3220 nm/RIU	57.5	-	[33]
Elliptical resonator	Ag	Biosensing	550 nm/RIU	282.5	304.06	[51]
Circular cavity	Au	Temperature	−0.44 nm/°C	-	-	[137]
Nanodot embedded in a circular cavity	Au	Temperature	−0.63 nm/°C	-	-	[137]
Circular cavity	Au	Temperature and biosensing	−0.336 nm/°C and 737.71 nm/RIU		20.5	[89]
Two stub and one ring resonator	Ag	Biosensing	1268 nm/RIU	280	-	[150]
Circular split ring	Ag	Biosensing	1250 nm/RIU	54	-	[151]
Racetrack integrated circular cavity	Au	Biosensing	1400 nm/RIU	12.01	-	[35]
Rectangular hollow cavity with a metallic island inside	Au	Tuberculosis in blood plasma	900 nm/RIU	11.84	-	[152]
Ring cavity with stub	Ag	Biosensing and temperature sensing	2010 nm/RIU and −0.90 nm/°C	49,219.04	-	[153]
Semicircular cavity	Au	Biosensing	941.33 nm/RIU	-	-	[21]
Arc resonator structure	Ag	Biosensing and gas sensing	867.2 nm/RIU and 142.1 pm/ppm	57.8	65.9	[154]
Circular cavity	Au	Biosensing	1132.14 nm/RIU	48.17	-	[155]
Taiji resonator	Ag	Biosensing	2016 nm/RIU	-	317	[156]
Modified Bragg grating	Au	Temperature	−0.47 nm/°C	-	-	[157]
Ohm-shaped and D-shaped resonators	Ag	Biosensing	4459.05 nm/RIU	297.67	-	[158]
Circular and rectangular cavity	Au	Biosensing and temperature	700 nm/RIU and −0.35 nm/°C	21.9 and 0.008	-	[149]
Square-shaped cavity	Au	Gas sensing	135.95 pm/ppm	-	-	[159]
U-shaped cavity with three stubs	Ag	Alcohol solution concentration	2900 nm/RIU	55.76	-	[54]
E-shaped cavity	Ag	Glucose and plasma	0.323 nm∙L/g and 0.243 nm∙L/g	-	-	[160]
Square ring resonator	Au	Gas sensing	1320 nm/RIU	16.7	-	[161]
Concentric double ring resonator	Ag	Biosensing and temperature sensing	2260 nm/RIU and 1.48 nm/°C	-	-	[130]
Semicircular ring rectangular cavity	Ag	Biosensing and temperature sensing	2560 nm/RIU and 0.87 nm/°C	10.8	-	[162]
Nanodots decorated asymmetric cavity	Ag	Biosensing	2464 nm/RIU	-	-	[69]
Nanorods embedded in square ring-shape	Ag	Biosensing	2473 nm/RIU	34.18	56.35	[65]
Modified ring resonator design	Au	Biosensing	1155.71 nm/RIU	25.9	-	[55]
Two T-shaped cavities	Ag	Pressure and temperature	12.48 nm/MPa and 0.36 nm/°C	-	-	[132]

## 7. Light-Coupling Mechanism to MIM WG

Coupling light to an MIM WG, where the size of the insulator (often referred to as an air slot) is smaller than the wavelength of light, presents significant challenges but also fascinating opportunities for innovative design and engineering solutions. The subwavelength dimensions of the air slot necessitate the use of methods that can effectively bridge the scale difference between free-space or conventional WG light and the tightly confined modes within the MIM structure. One common approach is to use a tapered structure, where the WG gradually narrows, easing the transition of light into the small air slot [53,152,155]. This tapering can be designed to adiabatically compress the mode size, ensuring efficient coupling by gradually matching the mode profiles of the input light and the MIM WG.

Another effective technique involves the use of grating couplers. Grating couplers can diffract incoming light at specific angles to match the propagation constant of the MIM WG modes [163]. By carefully designing the grating’s period and depth, it is possible to achieve phase matching and direct a significant portion of the incident light into the WG. This method is particularly useful for integrating MIM WGs with planar photonic circuits, enabling efficient coupling from external light sources or other WGs [164]. Additionally, plasmonic lenses or nano-antennas can be employed to focus and direct light into the narrow insulator region [165]. These devices leverage the principles of plasmonics to concentrate light into subwavelength volumes, making them ideal for coupling light into MIM WGs. The plasmonic structures can be designed to resonate at specific wavelengths, enhancing the local EM field and facilitating the transfer of light into the confined WG modes [166].

Last but not least, hybrid orthogonal couplers for MIM WGs represent a sophisticated approach to efficiently couple light into these subwavelength structures by exploiting the orthogonality of different mode profiles [167]. These couplers are designed to facilitate the transfer of light between distinct modes in a controlled manner, often between a fundamental mode in a conventional WG and a higher-order mode in the MIM WG [89]. In this section, tapered WG couplers and hybrid orthogonal couplers are discussed, both of which are considerably covered in the literature due to their proven effectiveness and versatility in photonic applications.

### 7.1. Tapered WGs

Advancements in the ability to tightly confine photons at the deep-subwavelength spatial level have the potential to significantly transform both scientific research and engineering applications. Overcoming various loss mechanisms and the inherent challenges of on-chip nanofabrication have been major obstacles in achieving efficient light coupling into highly compact nanofocusing devices within the field of on-chip nanophotonics. In previous studies on plasmonic sensors established on MIM WGs, the complex mechanism of light coupling within nanoscale MIM WG structures had largely been overlooked by researchers [168]. Transforming a dielectric mode into a plasmonic mode requires the use of a dielectric mode converter, making the tapered WG a crucial component in this process. This WG structure features a gradual change in its cross-sectional geometry along its length, transitioning from a wider input end to a narrower output end [55]. This innovative design enables various functionalities, such as mode conversion, mode matching, and signal coupling. By precisely tailoring the taper profile, the transmission of light between the dielectric WG and the MIM WG can be seamlessly achieved, resulting in an efficient conversion from the dielectric mode to the plasmonic mode [155].

**Figure 6:**
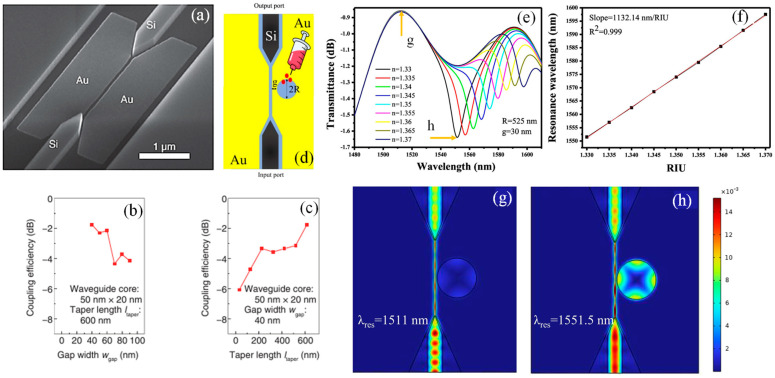
(**a**) SEM image of the mode converter [169], (**b**) CE of the mode converter versus gap width [169], (**c**) CE of the mode converter versus taper length [169], (**d**) graphical illustration of MIM WG- based plasmonic sensor integrated with Si tapered WG [155], (**e**) transmission spectrum of the device in the presence of different RI materials, (**f**) λ_res_ versus RIU, and norm. E-field pattern in the device in (**g**) off-resonance state [155] and (**h**) on-resonance state [155].

NTT developed a highly efficient plasmonic mode converter designed for the transition between a deep-subwavelength MIM WG, utilizing gold as the metal and air as the insulator, and a Si-wire WG (Figure 6a) [169]. The modes propagating through these two WGs differ significantly in both size and shape, necessitating a mode conversion that reduces size in both the lateral and vertical directions, thereby requiring 3D mode conversion. Previous studies addressed the lateral mode conversion between MIM and Si-wire WGs [118,170], but these methods only achieved 2D mode conversion, compressing solely in the lateral direction using a laterally tapered structure. Although a dual-tapered structure for both the vertical and lateral directions was proposed for 3D mode conversion [171], the complexity of its fabrication process renders it impractical for integration into optical circuits.

Figure 6b,c illustrate the CE of a fabricated mode converter for an MIM WG with a core size of 50 nm × 20 nm. The converter demonstrated a high coupling efficiency of –1.7 dB when the air gap width is 40 nm and the taper length is 600 nm, closely matching the calculated value of –1.4 dB. Additionally, the calculation results predicted the coupling efficiency dependence on various air gap widths and taper lengths, showcasing a strong correlation. Highly efficient mode conversion (–1.7 dB) between a deep-subwavelength MIM WG (50 nm × 20 nm core) and a Si-wire WG (400 nm × 200 nm core) was achieved using a mode converter with a taper length of 600 nm and an air gap width of 40 nm. Moreover, the core size of the connected MIM WG was (λ/n)^2^/2000 (where λ is the wavelength and n is the refractive index). This represented a significant reduction from the previously smallest core size of (λ/n)^2^/120 for lateral MIM WGs. Achieving highly efficient mode conversion with a taper length of less than 1 µm was crucial for minimizing the device footprint [169].

Kazanskiy et al. conducted a comprehensive numerical investigation of a RI sensor established on an MIM plasmonic WG [55]. The sensor employs a ring resonator design with a slight modification at the coupling segment, enhancing the efficiency of light coupling between a bus WG and the ring resonator, especially at the λ_res_. This deliberate variation drastically advances the device’s ER, a critical factor in its functionality. Amazingly, the sensor demonstrated an S of approximately 1155.71 nm/RIU and an FOM of 25.9. Additionally, the study explored the intricate mechanism of light injection into the nanoscale MIM WG. It was achieved through the integration of Si-tapered WGs, which facilitate the conversion of a dielectric mode into a plasmonic mode, and vice versa. After optimizing the geometry of the tapered WG for an operational wavelength of 1550 nm, further simulations over a broader wavelength range from 1400 nm to 1800 nm were conducted to assess its impact on CE, which remains consistently high, ranging from −2.39 dB to −5.41 dB across this wide spectral range [55].

Moreover, Butt et al. conducted a numerical evaluation to investigate the CE of mode converters for an MIM WG [155]. A dielectric-to-plasmonic mode converter in the form of a tapered WG to inject the light into the MIM WG and a plasmonic-to-dielectric mode converter to extract light from the plasmonic WG were designed. The CE of the 5000 nm long device was approximately −1.6 dB. Additionally, an MIM-WG-based sensor was devised by incorporating a circular hollow cavity for RI-sensing implementations, as shown in Figure 6d. The transmission spectrum versus the ambient RI is calculated in Figure 6e. It can be seen that a redshift was observed as the RI of the material under investigation increased. Moreover, the λ_res_ versus RIU trend is plotted in Figure 6f. The S and FOM of the device were 1132.14 nm/RIU and 48.17 RIU^−1^, respectively. The norm. E-field patterns in the sensor in an off-resonance state and on-resonance state are shown in Figure 6g and Figure 6h, respectively. It is believed that this research will pave the way for the development of favorably integrated plasmonic sensors established on MIM WGs [155].

### 7.2. Hybrid Orthogonal Coupling

Generally, the SPP mode experiences momentum mismatch with plane waves or conventional WG modes. To address this, prisms or Bragg gratings are typically used to couple plane waves to the SPP mode at a single interface, compensating for the momentum difference. The plasmonic slot mode, composed of two coupled SPP surface modes, confines TM light to a very small scale of a few tens of nanometers. This confinement makes it particularly challenging to use basic coupling techniques to interface with the plasmonic WG while maintaining practical CE. The weak coupling between the Si WG and the MIM WG primarily arises from a significant momentum mismatch between their fundamental modes. This mismatch is due to two main factors: first, the inherent momentum difference between the SPP mode at any single interface and the WG or plane waves, which necessitates a specialized coupling mechanism; second, the slot dimension of the MIM WG, which is typically an order of magnitude smaller than that of the conventional Si WG.

**Figure 7:**
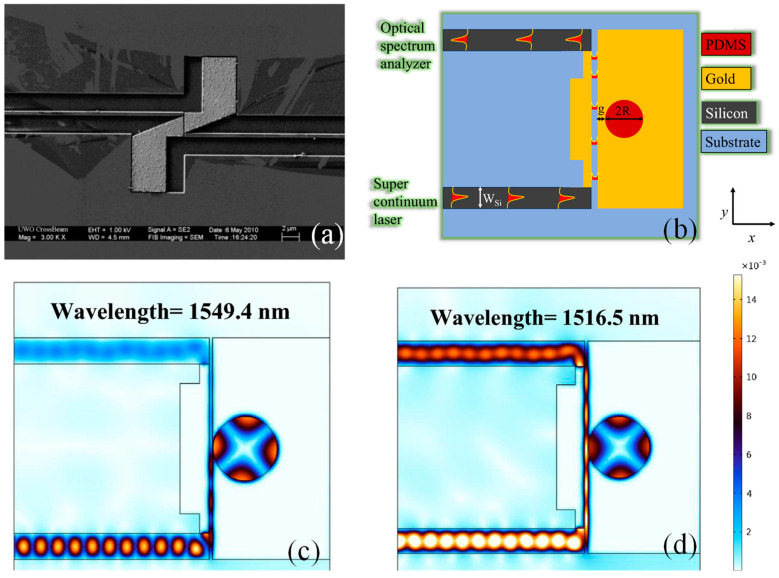
(**a**) SEM image of the orthogonal coupling configuration [167], (**b**) graphical depiction of a plasmonic sensor integrated with orthogonal mode couplers [137], (**c**) norm. E-field pattern in the device in an on-resonance state [137], and (**d**) norm. E-field pattern in the device in an off-resonance state [137].

A common method to reduce momentum mismatch involves using a tapered section to decrease the lateral dimension of the Si WG. This tapering technique has been applied in various designs [118,155,172]. However, this approach requires a long propagation section, which increases the overall size, losses, and complexity of the structure. Shortening the tapering section can lead to impedance mismatch at both ends, creating standing waves within the tapered section. These standing waves can cause resonance effects, thereby reducing the coupling bandwidth and compromising the WG’s performance. Achieving broadband coupling with minimal resonance effects is crucial. To meet these requirements, Lau et al. proposed an innovative orthogonal coupling scheme that employs a 90° angle between the plasmonic MIM WG and the Si WG, as illustrated in the SEM image of Figure 7a. This orthogonal coupling method offered another solution to momentum mismatch without relying on tapered sections. Instead of matching the longitudinal momentum component as in typical approaches, this scheme aligns the orthogonal momentum component of the Si WG *k*_*x*_ with the MIM WG momentum component, which enhances the CE.

Inspired by the hybrid orthogonal configuration, Butt et al. investigated a plasmonic temperature sensor established on an MIM WG integrated with orthogonal mode couplers [137]. This type of coupler works by aligning the modes of the dielectric and MIM WGs in orthogonal orientations, allowing for selective coupling to occur between modes with different effective indices. By engineering the coupler so that the electric fields of the two modes overlap in an optimized way, it enhances mode matching despite the inherent differences in propagation constants. The orthogonal configuration reduces the need for exact momentum matching, as it allows the dielectric mode to excite the MIM WG’s plasmonic mode efficiently by facilitating spatial overlap rather than direct phase matching. This enables the energy from the dielectric WG to couple into the plasmonic mode of the MIM WG with minimal reflection or scattering, overcoming the typical challenges posed by momentum mismatch and providing a pathway for efficient light transfer between the two distinct waveguiding structures.

The sensor featured a cavity covered with PDMS polymer, side-coupled to the MIM bus WG, as indicated in Figure 7b. The norm. E-field pattern in on-resonance and off-resonance states is illustrated in Figure 7c and Figure 7d, respectively. The device achieved an S of approximately −0.44 nm/°C, which can be improved to −0.63 nm/°C by adding a periodic arrangement of metallic nanoblocks in the cavity. Despite numerous sensitive plasmonic sensor designs, light-coupling mechanisms to nanoscale WGs remain underexplored. An innovative advancement is presented as follows: orthogonal mode couplers designed for plasmonic chips utilizing MIM-WG-based sensors. The hybrid system, optimized with Si couplers and an MIM WG, achieved transmission losses ranging from −1.73 dB to −2.93 dB over wavelengths spanning 1450–1650 nm. This integration of couplers represented a substantial enhancement for the plasmonic sensor, positioning it as an outstanding choice for a wide range of sensing applications.

## 8. Challenges Associated with the Development of MIM-WG-Based Sensors

Developing MIM-WG-based sensors presents a range of significant challenges, spanning materials science, fabrication techniques, and functional integration [168]. One of the primary challenges is related to the material properties of the metals and insulators used in these WGs. Metals like Ag and Al, commonly used due to their favorable plasmonic properties, are prone to oxidation and degradation over time, which can impair the sensor’s performance [173,174]. Additionally, the choice of the insulating material is critical; it must provide the right dielectric properties while maintaining compatibility with the metal layers. Materials such as silicon dioxide or polymers are often used, but their integration with metal layers without inducing significant losses or defects is complex and requires precise control over deposition techniques.

Another significant challenge is the fabrication process itself. MIM WGs require nanoscale precision in their construction, as even minor deviations in layer thickness or uniformity can lead to significant performance degradation. Advanced lithography techniques, such as electron-beam lithography (EBL) are often employed to achieve the required precision [175]. However, these techniques are not only costly but also time-consuming and limited by their throughput. Additionally, the alignment between the metal and insulator layers must be perfect to ensure optimal functionality, further complicating the fabrication process [176].

Moreover, the integration of MIM WG sensors with electronic and optical systems for signal readout and processing is a non-trivial task. This involves developing efficient coupling mechanisms to transfer the optical signals into and out of the WG without significant losses. It also requires designing compact and efficient electronics that can handle the high-frequency signals typically associated with plasmonic phenomena. Additionally, for practical applications, these sensors must be miniaturized and integrated into portable or wearable devices, which demands innovative packaging solutions to protect the delicate WG structures while maintaining their S.

Finally, the scalability and cost-effectiveness of producing MIM-WG-based sensors pose considerable challenges for widespread adoption. While these sensors offer high S and specificity, the sophisticated fabrication processes and the need for high-purity materials drive up production costs. Developing cost-effective manufacturing techniques that can be scaled up for mass production without compromising the sensor’s performance is crucial for its commercial viability. This necessitates ongoing research into novel fabrication methods, such as roll-to-roll processing or additive manufacturing, which can potentially offer solutions to the scalability issue.

The development of MIM-WG-based sensors involves addressing a multitude of challenges across material selection, fabrication precision, integration with functional systems, and scalable manufacturing. Overcoming these hurdles requires interdisciplinary collaboration and continued innovation in materials science, nanofabrication techniques, and sensor integration technologies. Despite these challenges, the potential applications of MIM-WG-based sensors in fields like medical diagnostics, environmental monitoring, and industrial sensing make them a highly promising area of research and development.

## 9. Concluding Remarks

MIM-WG-based sensors represent a significant advancement in the field of nanophotonics and plasmonics, offering unparalleled S and versatility for various sensing applications. The unique characteristics of MIM structures, such as their strong electromagnetic field confinement and enhanced light–matter interactions, position these sensors as highly effective tools for detecting minute changes in environmental conditions and analyte concentrations. The diversity in cavity shapes, including rectangular, circular, and more complex geometries, enables the customization of MIM sensors for specific applications. Each cavity shape influences the propagation and confinement of SPPs, allowing for the optimization of sensor performance parameters such as S, FOM, Q-factor, and LOD. This flexibility is crucial for tailoring sensors to meet the stringent requirements of various fields, including biomedical diagnostics, environmental monitoring, and chemical detection.

Material selection plays a pivotal role in the performance of MIM-WG-based sensors. The choice of metals, typically gold or silver, combined with dielectric materials like silicon dioxide or titanium dioxide, impacts the plasmonic resonance and overall sensor efficiency. Advances in materials science are continually expanding the range of available materials, leading to improved sensor robustness, biocompatibility, and integration capabilities with existing photonic circuits. Applications of MIM-WG-based sensors are vast and varied. In the biomedical sector, they are used for detecting biomolecules, pathogens, and other analytes with high S and specificity. Environmental monitoring benefits from the sensors’ ability to detect pollutants and hazardous substances at low concentrations. Additionally, these sensors are employed in chemical detection, providing the rapid and accurate identification of various chemical compounds.

Looking forward, the future perspective of MIM-WG-based sensors is promising. Continued research and development are expected to address current challenges, such as improving the fabrication techniques for better consistency and scalability, enhancing the hybrid integration with other photonic and electronic components, and developing more efficient light-coupling mechanisms. Overcoming these challenges will be crucial for the practical realization and widespread adoption of MIM sensors. Despite these advancements, several challenges remain, such as the hybrid integration of these sensing devices. The gap between numerical simulations and experimental results needs to be bridged, requiring more sophisticated modeling techniques and experimental setups. Furthermore, issues related to the stability and durability of the sensors under various operating conditions must be addressed to ensure their long-term reliability.

## Figures and Tables

**Table 1:** Essential attributes of MIM-WG-based sensors.

Attribute	Description
Structure	Consists of a thin insulating layer sandwiched between two metal layers.
Core Size	Can achieve deep-subwavelength dimensions, often on the nanometer scale.
Propagation Mode	Supports plasmonic modes, allowing light confinement below the diffraction limit.
Materials	Commonly uses metals like Au, Ag, and Al for the metal layers; uses air or dielectric materials for the insulator.
Mode Conversion	Requires 3D mode conversion due to differences in mode size and shape compared to traditional WGs.
Coupling Efficiency	High coupling efficiency with optimized air gap and taper length; typically, around −1.7 dB.
Sensitivity	High sensitivity due to strong field enhancement and confinement.
Application	Suitable for sensing applications, including biosensing and chemical detection.
Fabrication Complexity	Can be complex, especially for 3D taper structures; requires precise nanofabrication techniques.
Integration	Compatible with optical integrated circuits, though fabrication challenges exist.

## Data Availability

Not applicable.

## Abbreviations

MIM	Metal–insulator–metal
WG	Waveguide
SPP	Surface plasmon polariton
CE	Coupling efficiency
EM	Electromagnetic
PDMS	Polydimethylsiloxane
FOM	Figure of merit
LOD	Limit of detection
SPR	Surface plasmon resonance
FDTD	Finite difference time domain
FEM	Finite element method
PHMB	Polyhexamethylene biguanide
RIU	Refractive index unit
FWHM	Full width at half maximum
UV	Ultraviolet
